# Gene expression profiles in *BCL11B*-siRNA treated malignant T cells

**DOI:** 10.1186/1756-8722-4-23

**Published:** 2011-05-15

**Authors:** Xin Huang, Qi Shen, Si Chen, Shaohua Chen, Lijian Yang, Jianyu Weng, Xin Du, Piotr Grabarczyk, Grzegorz K Przybylski, Christian A Schmidt, Yangqiu Li

**Affiliations:** 1 Institute of Hematology, Medical College, Jinan University, Guangzhou, PR China; 2 Department of Hematology, Guangdong General Hospital (Guangdong Academy of Medical Sciences), Guangzhou, PR China; 3 Department of Hematology and Oncology, Ernst-Moritz-Arndt University Greifswald, Greifswald, Germany; 4 Institute of Human Genetics, Polish Academy of Sciences, Poznan, Poland; 5 Key Laboratory for Regenerative Medicine of Ministry of Education, Jinan University, Guangzhou, PR China

## Abstract

**Background:**

Downregulation of the B-cell chronic lymphocytic leukemia (CLL)/lymphoma11B (*BCL11B) *gene by small interfering RNA (siRNA) leads to growth inhibition and apoptosis of the human T-cell acute lymphoblastic leukemia (T-ALL) cell line Molt-4. To further characterize the molecular mechanism, a global gene expression profile of *BCL11B*-siRNA -treated Molt-4 cells was established. The expression profiles of several genes were further validated in the *BCL11B*-siRNA -treated Molt-4 cells and primary T-ALL cells.

**Results:**

142 genes were found to be upregulated and 109 genes downregulated in the *BCL11B*-siRNA -treated Molt-4 cells by microarray analysis. Among apoptosis-related genes, three pro-apoptotic genes, *TNFSF*10, *BIK*, *BNIP*3, were upregulated and one anti-apoptotic gene, *BCL2L*1 was downregulated. Moreover, the expression of *SPP*1 and *CREBBP *genes involved in the transforming growth factor (TGF-β) pathway was down 16-fold. Expression levels of *TNFSF*10, *BCL2L*1, *SPP*1, and *CREBBP *were also examined by real-time PCR. A similar expression pattern of *TNFSF*10, *BCL2L*1, and *SPP*1 was identified. However, *CREBBP *was not downregulated in the *BLC11B*-siRNA -treated Molt-4 cells.

**Conclusion:**

*BCL11B*-siRNA treatment altered expression profiles of *TNFSF*10, *BCL2L*1, and *SPP*1 in both Molt-4 T cell line and primary T-ALL cells.

## Background

Although treatment outcome in patients with T-cell acute lymphoblastic leukemia (T-ALL) has improved in recent years, relapsed T-ALL remains a challenge [1]. Monoclonal antibodies, gene inhibitors, and upregulation of microRNAs [2,3] are promising tools for cancer targeted therapy. However, few targeted therapies are available for T-cell malignancies. For example, transforming *Mer *signals may contribute to T-cell leukemogenesis, and regulation of *Mer *expression could be a novel therapeutic target for pediatric ALL therapy [4]. The recent identification of activating Notch1 mutations in the majority of patients with T-ALL has brought interests on targeting the Notch signaling pathway for this disease [5].

The B-cell chronic lymphocytic leukemia (CLL)/lymphoma 11B (*BCL11B*) gene was first identified on human chromosome 14q32.2 [6] and encodes a Krüppel-like C_2_H_2 _zinc finger protein initially identified as a transcriptional repressor [7]. *BCL11B *plays an important role in T-cell differentiation and proliferation [8-11]. Altered expression, mutation, disruption, or rearrangement of *BCL11B *has been associated with T-cell malignancies [12-14]. In humans, *BCL11B *overexpression is found primarily in lymphoproliferative disorders, such as T-ALL and adult T-cell leukemia/lymphoma [12,15-17]. *BCL11B *mediates transcriptional activation by interacting with the p300 co-activator at the upstream site 1 (US1) of the interleukin (IL)-2 promoter, leading to transcriptional activation of IL-2 expression in activated T cells [18]. Although the interaction partners and binding sequence have been revealed, only a few *BCL11B *direct target genes have been identified to date. Our previous study in the human T-ALL cell lines Molt-4, Jurkat, and hut78 has shown increased apoptosis upon *BCL11B *suppression by RNA interference [19].

In the present study, we further analyzed the global gene expression profiles in Molt-4 and primary T -ALL cells after *BCL11B*-935-siRNA treatment.

## Methods

### Samples

Samples from three newly diagnosed patients with T-ALL and one patient with T-cell lymphoma/leukemia were obtained after informed consent. The diagnosis of T-ALL was based on cytomorphology, immunohistochemistry, and flowcytometry analyses. The samples were named P1 (55-year-old male with T-ALL), P2 (6-year-old male with T-ALL), P3 (55-year-old female with T-cell lymphoma/leukemia), and P4 (19-year-old male with T-ALL). Peripheral blood was collected with heparin and peripheral mononuclear cells (PBMCs; contained more than 70% leukemic T cells) were separated using the Ficoll-Hypaque gradient centrifugation method. All procedures were conducted in strict accordance with the guidelines of the Medical Ethics committees of the Health Bureau of Guangdong province, China.

### Cell culture

Molt-4 cells (Institutes for Biological Sciences Cell Resource Center, Chinese Academy of Sciences, Shanghai, China) and PBMCs collected from the four patients were cultured in complete RPMI 1640 medium with 15% fetal calf serum and were maintained in a sterile incubator at 37°C, 95% humidity, and 5% CO_2_.

### Nucleofection

*BCL11B*-siRNA935 (Chinese patent application number: 200910193248.3) and the scrambled non-silencing siRNA control (*BCL11B*-sc) were designed with online software http://www.invitrogen.com and synthesized by Invitrogen (Carlsbad, CA, USA).

Malignant T cells were resuspended at 2.5 × 10^6 ^(Molt-4 cells) or 1 × 10^7 ^(PBMCs) per 100 μL of the appropriate Nucleofector kit solution (Amaxa Biosystems, Cologne, Germany), and were nucleofected with 3 μg of BCL11B-siRNA or control non-silencing scrambled (sc) RNA using the C-005 (Molt-4 cells) or U-014 (PBMCs) program in the Nucleofection Device II (Amaxa Biosystems). Mock-transfected cells (nucleofected without siRNA) were used as a negative control. After nucleofection, the cells were immediately mixed with 500 μL of pre-warmed culture medium and transferred to culture plates for incubation. Samples were collected for RNA isolation.

### RNA isolation, expression profiling, reverse transcription, and real-time PCR

Total RNA was isolated using Trizol (Invitrogen), and cDNA was synthesized with a Superscript II RNaseH Reverse Transcriptase kit (Invitrogen).

Total RNA (> 3 μg) was sent for global gene expression profile analysis using an Affymetrix HG U133 Plus 2.0 gene chip (Shanghai Biochip Co., Ltd., Shanghai, China). The Affymetrix microarray analysis was performed using Gene Spring GX10.0 software (Agilent Technologies, Santa Clara, CA, USA).

The primer and probe information for *BCL11B *and the reference gene β-2-microglobulin (*β2-MG*), as well as the details of the real-time PCR for *BCL11B *were described in our previous studies [12,15]. Expression levels of tumor necrosis factor (ligand) superfamily, member 10 (*TNFSF*10; *TRAIL*), *BCL*2-like 1 (*BCL2L*1; *Bcl-xL*), secreted phosphoprotein 1 (*SPP*1), cAMP-response element binding protein (*CREBBP*), and *β2-MG *were determined by real-time PCR using a SYBR Green I qPCR Master Mix kit [15].

### Flow cytometry assay

Cells from different groups were prepared according to the protocols, and the *BCL2 *expression level was measured by flow cytometry (Beckman Coulter, Fullerton, CA, USA). Mouse anti-human *BCL2*-PE and mouse IgG1-PE (eBioscience, San Diego, CA, USA) were used. Results were analyzed using the Win MDI 2.9 software.

## Results and discussion

### Global gene expression profile in *BCL11B*-siRNA935 treated Molt-4 cells

**Figure 1:**
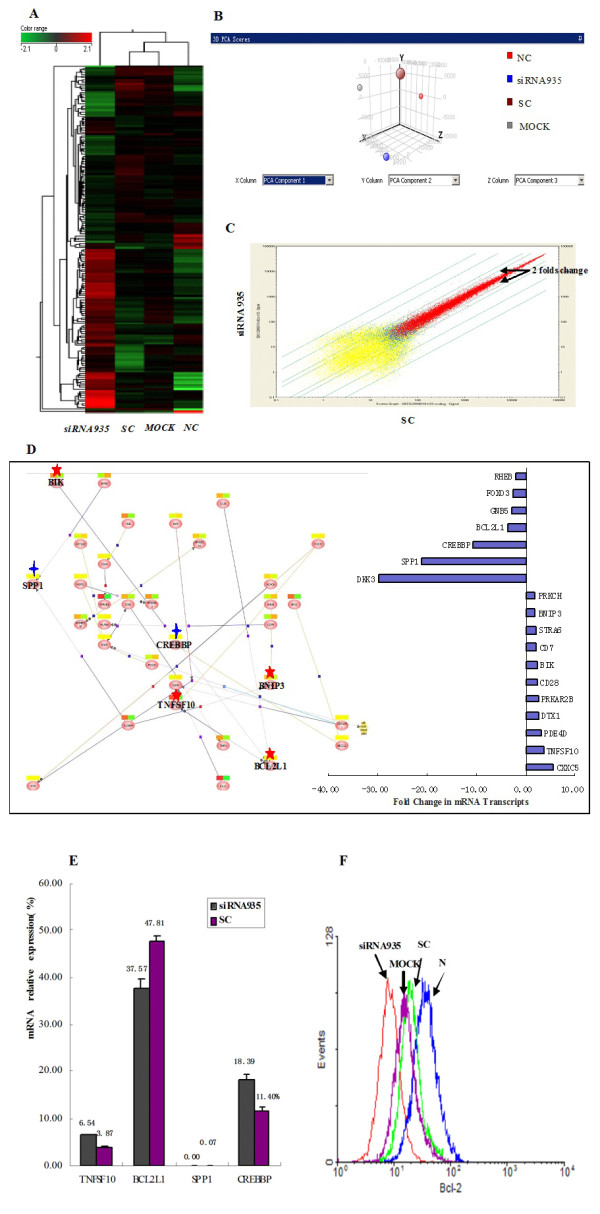
**Results of the gene chip microarray analysis and validation**. (A) Visual display of the cluster analysis for the *BCL11B *siRNA935-transfected and control cells. (B) Principal component analysis. The closer the dots, the more similar the gene expression profiles are; the farther apart the dots, the greater the differences are. (C) Two-dimensional scatterplot analysis of gene expression values for all genes on the *BCL11B *siRNA935-transfected cells and control cells from the microarray. Yellow dots represent genes absent from both samples; blue dots represent genes present in one sample but absent from the other sample; and red dots represent genes present in both samples. Dots outside the 2 × difference lines, indicated by black arrows, represent differentially expressed genes. The farther from the line, the greater the difference in gene expression are. (D) Analysis of pathways closely related to tumor cell proliferation and apoptosis. Results are shown as fold-change in mRNA transcripts. Genes indicated with a red star are in the apoptosis pathway; genes indicated with a blue star are in the transforming growth factor-β pathway. (E) Gene validation by real-time PCR. Changes in *TNFSF*10, *BCL2L*1, and *SPP*1 expression levels agreed with the microarray results, while those of *CREBBP *did not. (F) Reduced *BCL*-2 protein expression was confirmed by flow cytometry. *BCL*-2 expression in *BCL11B *siRNA3-transfected cells was significantly lower, at 46% of that in SC (99.1%), MOCK (99.2%), and NC cells (99.7%).

To determine the molecular mechanisms of *BCL11B *siRNA-mediated cell apoptosis, global gene expression profiling was performed at 24 h post-transfection, when *BCL11B *mRNA was most effectively suppressed (data not shown). Results were clustered, based on the differential expression level (2-fold up or down), and visualized using a color scale (Figure 1A). Principal component analysis indicated that the changes in the Molt-4 cell gene expression profile could be accounted for primarily by the *BCL11B *siRNA935 treatment (Figure 1B). A GCOS1.4 software analysis showed that upregulated genes were identified by 142 probe sets, whereas 109 genes were downregulated at least 2-fold, compared with the sc control (Figure 1C). Changes in genes of the same signaling pathways closely related to tumor cell proliferation and apoptosis were analyzed further (Figure 1D).

**Figure 2:**
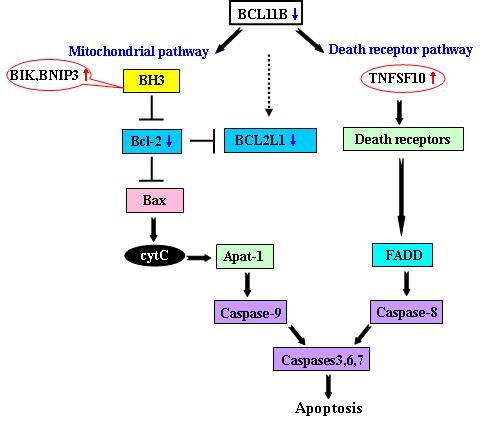
**Schematic model of the molecular mechanism of *BCL11B*-siRNA-mediated apoptosis in Molt-4 cell [modified from reference 20]**.

Among apoptosis-related genes, changes in expression levels occurred mainly in three pro-apoptotic genes; *TNFSF*10, *BCL*-2 interacting killer (*BIK*), and *BCL*-2/*E1B *19 kDa interacting protein 3 (*BNIP*3), which were upregulated 2-4 fold, and one anti-apoptotic gene (*BCL2L*1) was downregulated by 3-4 fold. The expression levels of *SPP*1 and *CREBBP *genes involved in the transforming growth factor (TGF-β) pathway were down by 16 fold. The changes in the expression levels of the *TNFSF10*, *BCL2L*1, *SPP*1, and *CREBBP *genes were further detected by real-time PCR (Figure 1E). The BH3-only domain proteins BIK and BNIP3, which were located upstream of BCL-2 (Figure 2), may enhance their binding to BCL-2, thereby inhibiting the anti-apoptotic function. Thus, we analyzed the *BCL-2 *protein expression level by flow cytometry in Molt-4 cells at 72 h after *BCL11B*-siRNA treatment (Figure 1F). A similar altered expression pattern of these genes, as well as expression of the *BCL-2 *protein, was confirmed. However, *CREBBP *did not show downregulation in BCL11B-siRNA treated Molt-4 cells.

The global gene expression profile results suggest that the molecular mechanisms of *BCL11B *siRNA-mediated cell death may involve *BCL*-2 family genes in the intrinsic mitochondrial pathway as well as the *TNFSF*10 gene in the death receptor signaling pathway (Figure 2) [20]. Upregulation of the *TNFSF*10 gene activated the death receptor signaling pathway, whereas upregulation of the two mitochondrial *BCL*-2 family genes (the BH3-only domain proteins *BIK *and *BNIP*3) enhanced their binding to *BCL*-2, with a reduction in the anti-apoptotic gene *BCL2L*1, thereby inhibiting the anti-apoptotic function and promoting *Bax *and *Bak *activation. This in turn activates the downstream caspases 3, 6, and 7, leading to increased apoptosis. Reduced expression of *SPP*1 correlated with increased apoptosis in Molt-4 cells, suggesting that the *SPP*1 gene may be a *BCL11B *gene target.

**Figure 3:**
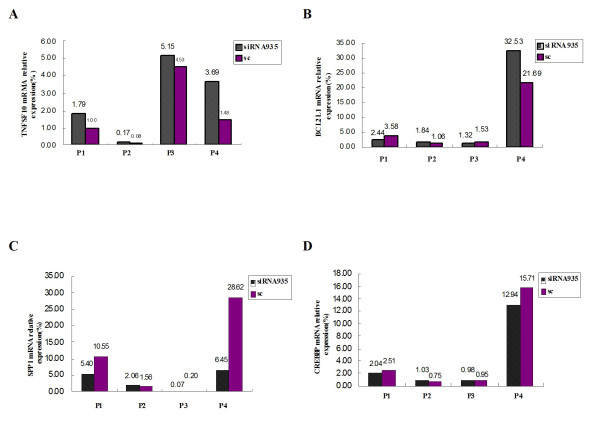
**Expression of *TNFSF*10, *BCL2L*1, *SPP*1, and *CREBBP *genes in peripheral mononuclear cells from four patients (P1-P4) with T-cell acute lymphoblastic leukemia at 24 h after *BCL11B *siRNA transfection**.

*CREBBP *overexpression has been detected in Jurkat cells [21]. However, previous studies have not reported a change in *CREBBP *expression in T cell lines after BCL11B-siRNA treatment. In the present study, downregulation of *CREBBP *was identified in the microarray analysis, but not confirmed by real-time PCR analysis. The reason may be due to a systemic error on the microarray analysis. Interestingly, unlike the result from Molt-4 cells, the alteration of the *CREBBP *expression level in primary T-All cells after *BCL11B*-siRNA treatment was in accordance with the results from the microarray analysis (Figure 3). Thus, the role of *CREBBP *during *BCL11B *downregulation in malignant T cells requires further investigation.

### Expression of *TNFSF*10, *BCL2L*1, *SPP*1, and *CREBBP *genes in *BCL11B*-siRNA935-treated primary leukemic T cells

After obtaining interesting data from Molt-4 cells, we analyzed the effect of the *BCL11B*-siRNA in primary T-ALL cells. We examined the expression levels of *TNFSF*10, *BCL2L*1, *SPP*1, and *CREBBP *in primary leukemic T cells after *BCL11B *siRNA935 treatment. *BCL11B *expression level decreased in primary leukemic T cells treated with *BCL11B *siRNA935 (282.77 ± 247.57 copies/10^5 ^*β2-MG*) as compared with the sc control group (519.48 ± 303.41 copies/10^5 ^*β2-MG*). The *TNFSF*10, *BCL2L*1, *SPP*1, and *CREBBP *expression levels in *BCL11B*-siRNA935- treated primary leukemic T cells were 2.7 ± 2.17%, 9.53 ± 15.34%, 3.5 ± 2.95%, and 4.25 ± 5.82%, respectively, whereas the expression levels in primary leukemic T cells in the sc control group were 1.77 ± 1.93%, 6.96 ± 9.88%, 10.23 ± 13.09%, and 4.98 ± 7.2%, respectively. The T-ALL specimen number was too small to perform statistical analysis. The changes in the mRNA levels of *TNFSF*10, *SPP*1, and *CREBBP *in the T cells from the four patients agreed in general with those from the microarray analysis results (Figure 3A, C, D). However, the changes in the *BCL2L*1 expression levels in the different samples varied (Figure 3B). The reduced *BCL2L*1 expression rates in leukemic T cells from patients 1 and 3 were 31.84% and 13.73%, respectively, compared with the sc controls, whereas *BCL2L*1 expression in leukemic T cells from patients 2 and 4 was upregulated. Although *BCL11B *gene overexpression occurred in all samples, it may have been due to the heterogeneity of T-cell malignancies during apoptosis induced by *BCL11B *downregulation [22], so it remains to be determined whether apoptosis induced by *BCL11B *downregulation in some cases with T-ALL involves the *BCL*-2 family genes in the intrinsic mitochondrial pathway.

A previous analysis revealed that overexpression of the *BCL11B*, *BCL2L*1, and *CREBBP *genes in primary T-ALL samples blocks apoptosis in malignant T cells [15]. This study suggests that inhibition of *BCL11B *may trigger apoptosis in leukemic T cells by downregulating the downstream genes *SPP*1, *CREBBP*, and *TNFSF*10.

## Conclusions

Our findings provide evidence that *BCL11*B siRNA-mediated cell apoptosis may be related to the mitochondrial pathway *BCL*-2 family genes and the *TNFSF*10 gene of the death receptor signaling pathway. Moreover, the *SPP*1 and *CREBBP *genes in the TGF-β pathway may also be involved in *BCL11B *siRNA-mediated cell apoptosis.

## Competing interests

The authors declare that they have no competing interests.

## Authors' contributions

YQL contributed to concept development and study design. XH, QS, SC, SHC, LJY performed the laboratory studies. PG, GKP and CAS provided some materials and technical support. JYW and XD were responsible for collection of clinical data. YQL and XH coordinated the study and helped drafting the manuscript. All authors read and approved the final manuscript.
